# Regulatory roles of R2R3-MYB genes in plant growth, development and stress adaptation: insights into seed dormancy and germination

**DOI:** 10.1007/s00425-026-05048-1

**Published:** 2026-07-04

**Authors:** Angel J. Matilla, Javier Fuertes-Aguilar

**Affiliations:** 1 Departamento de Biología Funcional, Universidad de Santiago de Compostela, 14971 Santiago de Compostela, Spain; 2 Real Jardín Botánico (CSIC), Plaza de Murillo 2, 28014 Madrid, Spain

**Keywords:** Agronomic crops, Anti-inflammatory, Diversification, Dormancy and germination, GAMYB R2R3-TF, Immunity, Phenylpropanoids, Phytohormones, Seed-coat pigmentation, TT1 and TT2

## Abstract

**Main conclusion:**

GAMYBsand TT2 are R2R3-MYB TFs involved in seed dormancy and germination, with GAMYBs mediating GA signaling and TT2 regulating seed-coat proanthocyanidins.

**Abstract:**

The MYB (v-myb avian myeloblastosis viral oncogene homolog) transcription factors (TFs) constitute one of the most diverse and evolutionarily conserved families of plant regulatory proteins, contributing to vegetative reproduction, anthocyanine (ANC) and triterpenoids biosynthesis, abiotic stress responses, immunity process and plant diversification. MYB proteins are ancient regulators, originating approximately one billion years ago in early eukaryotes and subsequently diversifying in plants and animals. The expansion of MYB genes in plants underlies their key role in generating phenotypic variation. Among the existing MYB TFs, the R2R3-MYB subfamily has undergone extensive expansion and diversification in land plants, conferring broad regulatory versatility across developmental, metabolic, and stress-response processes. Collectively, R2R3-MYB TFs are closely linked to phytohormone signalling and play a central role in fine-tuning of plant response networks. Additionally, R2R3-MYB and GAMYB TFs play central and multifaceted roles in regulating seed development, seed-coat pigmentation, dormancy, and germination. Members of the R2R3-MYB, such as MYB56, MYB62, MYB96, and MYB30, and GAMYB-related TFs highlighted here, integrate hormonal cues (ABA, GA, JAs, and NO) with transcriptional networks governing flavonoid biosynthesis, seed size determination, reserve mobilization, and seed-coat architecture. Through transcriptional and post-translational regulation, these TFs balance dormancy maintenance and germination competence. The regulatory roles of R2R3-MYB and GAMYB TFs during the final phase of the seed life cycle (SLC) are reviewed, as this topic has received little attention to date. Notably, this review synthesizes recent advances on the structural, evolutionary, and functional roles of GAMYB and TT2 TFs, including a phylogenetic analysis that highlights lineage-specific gene ascription, conserved clades, and evolutionary relationships within the R2R3-MYB family. Finally, perspectives and challenges are summarized to inform future studies on these important TFs in seed dormancy and germination.

## Updated background

One of the most important evolutionary advances in higher plants was the emergence of the seed—a specialized propagule essential for plant dispersal, survival, and reproductive success in terrestrial environments (Bai et al. 2022; Leubner 2024; Matilla 2025). As reproductive units, seeds ensure the transmission of genetic material between generations and play a central role in the plant life cycle. They allow plants to survive unfavorable conditions, disperse across diverse habitats, and coordinate germination with optimal environmental cues.

The seed life cycle (SLC) comprises several interconnected phases, including seed development and maturation, dormancy, germination, and seedling establishment (Sripathy And Groot 2023; Otani et al. 2024; Sajeev et al. 2024; Zhao et al. 2024a; He et al. 2025; Matilla 2025). Each stage is tightly regulated by a complex network of genetic and molecular mechanisms. These involve internal signals—such as phytohormones and TFs—as well as epigenetic modifications and environmental inputs including light, temperature, water availability, and abiotic stresses (such as drought, salinity, cold, etc.).

Recent studies have emphasized the dynamic interplay among hormonal pathways, reactive oxygen species (ROS), and chromatin-level regulation in controlling seed behavior. In particular, the opposing balance between ABA and GAs plays a central role in modulating seed dormancy and germination. These hormonal signals, together with ROS acting as secondary messengers and chromatin modifications, fine-tune seed responses to both internal developmental cues and external environmental conditions (Matilla et al. 2015; Li et al. 2022a; Nogueira do Amaral et al. 2024; Zhao et al. 2024a; Wang et al. 2025a). For instance, gamma-aminobutyric acid (GABA) has been shown to promote dormancy release—at least in *Arabidopsis*—by upregulating *GA20ox1*; downregulating *NCED6*; and altering ABA catabolism via *CYP707A2* transcription. GABA also modulates downstream signaling via interactions with DELLA proteins and ABI3 (Wang et al. 2025c). Other phytohormones, such as auxin, brassinosteroids (BRs), ethylene, and Jas, also play crucial roles in modulating different phases of the SLC through their interactions with these two key hormones (Liu et al. 2013; Iglesias-Moya et al. 2023; Corbineau 2024; Ma 2025; Nikolic et al. 2025). Recently, it has been shown that during the germination of *A. thaliana*, auxin contributes to the synergistic modulation of ABA signaling by JAs (Mei et al. 2023).

In addition to hormonal regulators previously mentioned, the Delay of Germination 1 (*DOG1*) gene is a key genetic determinant of seed dormancy. First identified in Arabidopsis as a major quantitative trait locus (QTL), mutations in *DOG1* result in reduced dormancy and premature germination (Bentsink et al. 2006; Carrillo-Barral et al. 2020; Xiang 2022). In wheat, *TaDOG* genes exhibit distinct hormonal responses and tissue-specific expression, underscoring their multifaceted roles in seed dormancy, development, and stress adaptation (Ni et al. 2025). More recently, *AhDOG1-3* has emerged as a promising molecular candidate for modulating seed dormancy across a broad range of plant species (Chen S. 2025, personal communication). Moreover, functional knockout of *CsDOG1* resulted in high susceptibility to preharvest sprouting (PHS), confirming its critical role in PHS regulation (Cao et al. 2025).

As described above, TFs play pivotal roles in controlling the SLC by modulating the expression of hormone-responsive and developmentally controlled genes involved in hormone biosynthesis, signaling, and response pathways. Despite this, our understanding of TFs in the spatio-temporal and hormone-mediated regulation of seed development, dormancy, and germination remains limited. In particular, ABA, a key hormone involved in the induction and maintenance of seed dormancy, is known to interact with multiple TFs. However, the precise mechanisms by which ABA signaling modulates their activity—such as through changes in expression patterns, post-translational modifications, or interactions with other regulatory proteins—are not yet fully understood. Recent studies have begun to address some of these gaps. For spatial transcriptomic analyses of ABA-regulated gene expression in germinating barley embryos have revealed region-specific expression patterns in response to this sesquiterpene phytohormone (Sybilska et al. 2025). Similarly, recent work in *Pyrus betulaefolia* has shown that seed coat-derived ABA regulates dormancy by modulating the balance of ABA and GA, implicating specific TFs, such as ABI5, in this hormonal interplay (Wang et al. 2025a).

Similar uncertainties also apply to GA. Although several TFs have been implicated in GA signaling pathways, the detailed mechanisms by which these diterpenoid hormones influence their regulatory functions—particularly during the transition from dormancy to germination—remain poorly understood. Recent studies have begun to address some of these gaps. For example, Luo et al. (2024) showed that PHYTOCHROME-INTERACTING FACTOR4 (PIF4) interacts with ABI4 to form a transcriptional activator complex that promotes ABA biosynthesis and signaling, thereby enhancing primary seed dormancy (Luo et al. 2024). Together, these findings highlight both the progress made and the remaining gaps in our understanding of how ABA and GA interact through TFs, to regulate seed dormancy and germination.

In conclusion, multiple TF families play central roles in regulating SLC processes. ABA-associated TFs, such as ABI5 (Kim et al. 2014), reinforce seed dormancy, whereas GA-related TFs, including bHLH PIFs, promote germination by counteracting ABA signaling. Moreover, NAC, and WRKY families integrate hormonal and environmental cues to coordinate developmental transitions. Recent studies highlight the importance of TF-mediated networks in hormonal crosstalk, stress responses, and seed development, with NAC (NAM, ATAF1/2, and CUC2) TFs family playing key roles in dormancy and germination regulation (Fuertes-Aguilar et al. 2024).

Within this framework, MYB TFs have emerged as major regulators. Originating ~ 1 billion years ago (Lipsick 1996), they have diversified extensively, acquiring a wide range of functions. This review summarizes recent advances in the physiological, molecular, and phylogenetic characterization of MYB proteins, particularly R2R3-MYB members involved in seed dormancy and germination. In particular, the regulatory roles of R2R3-MYB TFs, including the GAMYB subgroup, during the final phase of the seed life cycle (SLC) are highlighted, as this topic has received limited attention to date. Together, these findings provide a strong foundation for future research on the regulatory networks controlling seed behavior.

## Structural features and evolutionary dynamics of MYB TFs

The first MYB genes cloned in plants (e.g. the *C1* gene from maize) showed strong structural homology to the vertebrate cellular proto-oncogene c-MYB (Martin et al. 1997). Early investigations of MYB TFs revealed that plants contain a much larger number of MYB genes than animals, as well as many pairs of paralogous genes, which indicates an expansion and diversification of the family in the plant lineage. This diversification allows MYB genes to play diverse roles in numerous physiological and biochemical processes (Romero et al. 1998). In addition, recessive alleles of the maize C1 gene lead to the absence of anthocyanin (ACN) in the embryonic organs of the grain (Paz-Ares et al. 1986, 1987).

**Fig. 1:**
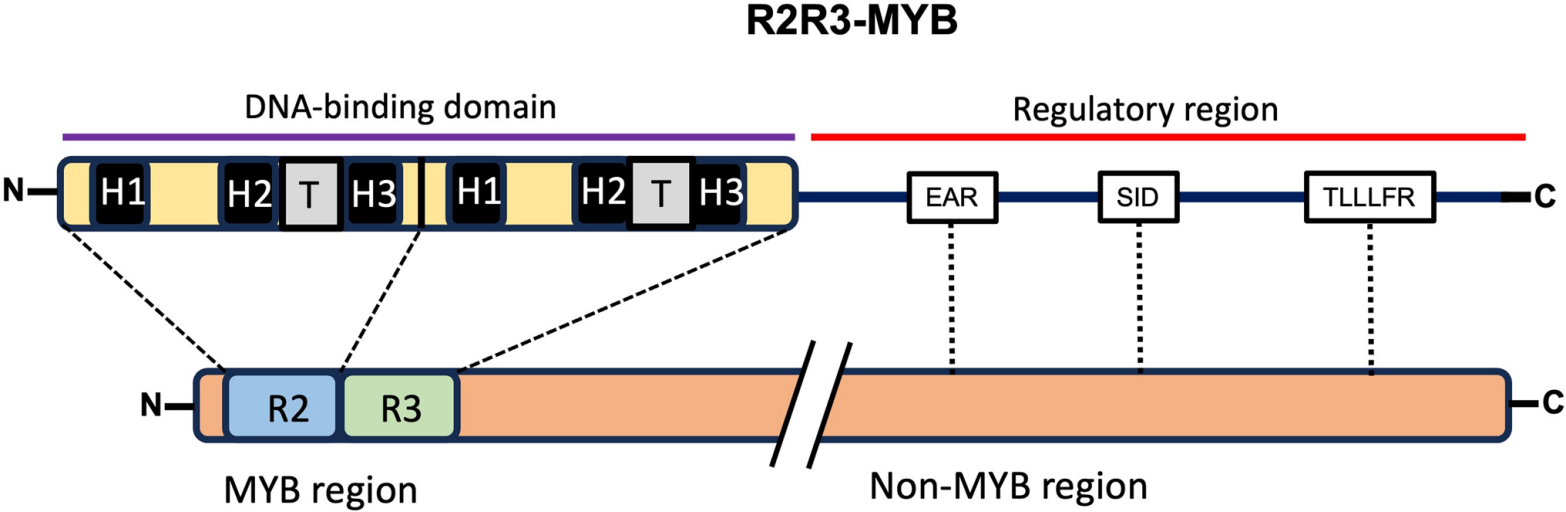
Structure and functional characterization of an R2R3-MYB transcription factor. This TF contains conserved R2 and R3 repeats, while the C-terminal region often shows divergence and may include one or more typical repressor motifs, such as the EAR (ERF-associated amphiphilic repression motif), SID (Sensitive to ABA and Drought 2 protein interaction motif), and TLLLFR. These three motifs are not present in all members of this TF family. H, α-helix; T, loop; a short connecting segment between two helix. The shaded bar represents the number of R2R3-MYBs with unknown (i.e., not experimentally validated) functions

The publication of *Arabidopsis* genome sequence allowed for the first comprehensive classification of MYB genes in plants (Stracke et al. 2001). Structurally, TFs typically contain DNA-binding and transcriptional regulatory domains, oligomerization sites, and nuclear localization signals. In plants, MYB TFs are characterized by a relatively conserved MYB domain located at the N-terminal region of the protein (Dubos et al. 2010; Wu et al. 2024) (Fig. 1). This conserved DNA-binding domain (DBD) mediates DNA binding and is classified according to the number of R repeats it contains.

**Fig. 2:**
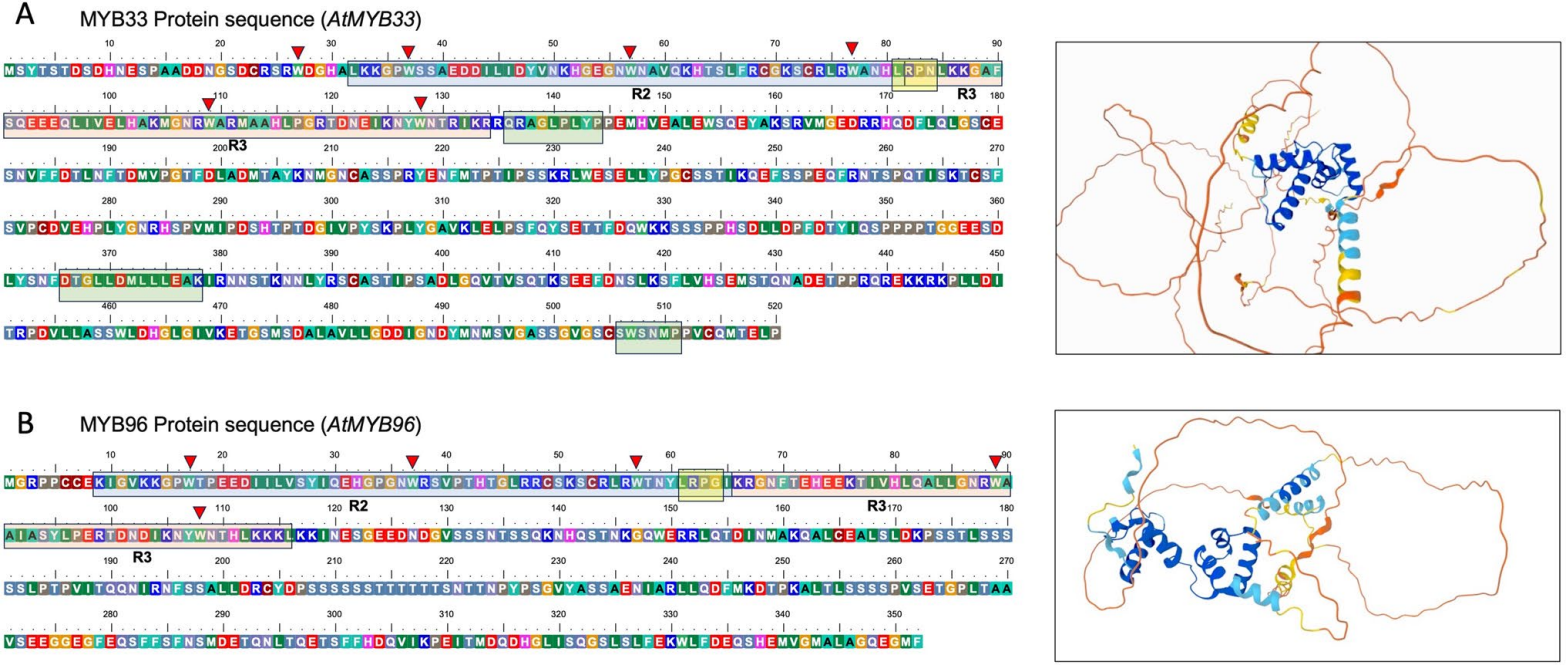
Comparative sequence analysis of AtMYB33 (**A**) and AtMYB96 (**B**) R2R3-MYB proteins from *Arabidopsis thaliana*, showing their complexity and heterogeneity within a single species: (i) the R2 and R3 domains contain tryptophan residues (W), indicated by red triangles, which contribute to stabilizing the α-helix conformation; (ii) the yellow box indicates a short linker connecting the R2 and R3 helices and providing a certain degree of structural flexibility; (iii) green boxes mark regions involved in biological processes, including response to ABA and cell differentiation, as well as pollen development, GA-mediated signaling, positive regulation of ABA signaling, and programmed cell death. The predicted three-dimensional models (right) for AtMYB33 and ATMYB96 reveal the characteristic α-helical MYB fold in the central region, flanked by likely intrinsically disordered N- and C-terminal regions. The heterogeneity between both sequences, A and B, is reflected, among other structural features, by the absence of green boxes in AtMYB96, likely due to differences in regulatory processes compared to those described for AtMYB33, as a result of functional and evolutionary adaptation

Each repeat, composed of approximately 51–52 amino acid residues, fits into the major groove of double-stranded DNA adopting a helix–turn–helix (HTH) conformation (Fig. 2). The repeat folds into a three–α-helix bundle stabilized by a cluster of conserved tryptophan (W) residues that form a hydrophobic core. Within this arrangement, the second and third helices constitute the canonical HTH motif, whose structural stability is reinforced by regularly spaced W or other hydrophobic residues (e.g. Phe or Leu) (Ogata et al. 1996). The third helix is considered to be the ‘recognition helix’ and is responsible for recognizing the DNA binding site and structurally binding to the target DNA in the major groove (Ogata et al. 1996; Yang et al. 2022). Each R repeat can function synergistically in DNA binding (particularly in R2) or individually in protein–protein interactions (Feller et al. 2011). In contrast, the C-terminal region is considerably more variable and enriched in acidic amino acids, which enables functional specialization, specific interactions, and diversification of regulatory mechanisms (Kranz et al. 1998).

R2R3-MYB genes in Arabidopsis are numerous, but show no clear genomic organization, as they are neither clustered nor arranged in simple groups. The number of R2R3-MYB transcription factors increased throughout green plant evolution, from fewer than 12 in chlorophytes to higher numbers in streptophytes and land plants (Feller et al. 2011; Bowman et al. 2017). Most land plant R2R3-MYBs lack orthologs in algae, with the exception of FLP/AtMYB124 and DUO1/AtMYB125 (Higo et al. 2018). Despite insights from algal genome sequencing, the early evolution of R2R3-MYBs before the colonization of land remains poorly understood.

In addition to their evolutionary expansion and diversification, expression data indicate that many R2R3-MYB genes display distinct patterns, consistent with their roles in diverse regulatory processes. At the end of the last century, three subtypes of R2R3-MYB proteins in *Arabidopsis thaliana* were described, namely A, B, and C. Subtype A (~ 10%) includes genes without any intron in the sequenced region; subtype B (~ 5%) contains genes with an intron at position 3; and subtype C (~ 85%) includes genes with an intron at position 2 (Romero et al. 1998).

On the other hand, due to potential redundancy among similar genes of R2R3-MYBs family, many specific functions remain unclear or have only recently been investigated (Yuhazu et al. 2024; Kranz et al. 1998). The extensive expansion of this family in plants compared to animals suggests that R2R3-MYB genes, which are hypothesized to have lost the first R-repeat (Rosinski et al. 1998), have played a key role in the evolution of plant-specific processes (i.e. rapid expansion of the R2R3-MYB TF family) (Martin et al. 1997).

Interestingly, R2R3-MYB TFs have greatly expanded in land plants compared to algae, while the composition of major subfamilies has remained largely conserved (Jiang et al. 2020). This expansion was driven by both whole-genome and segmental duplications, leading to extensive diversification of specific subfamilies across bryophytes, lycophytes, ferns, gymnosperms, and angiosperms. Conserved intron–exon structures and characteristic C-terminal motifs suggest that gene structure and protein domains contributed to functional specialization. As a result, R2R3-MYB genes play central roles in plant-specific processes, such as secondary metabolism, tissue development, pigmentation, and responses to biotic and abiotic stress (Jiang et al. 2020).

Thus, plant MYB proteins are classified into three major groups. The first and most common group is the R2R3-MYB subfamily, which is abundant in both monocots and dicots. The number of R2R3-MYB genes in plant genomes ranges from about 50 to over 400. For example, *Brassica napus* contains ~ 425 R2R3-MYB genes (Hajiebrahimi et al. 2017), *Triticum aestivum* (wheat) 393 genes (Zhang et al. 2012); *Glycine max* (soybean) 244 genes (Du et al. 2012), *Populus trichocarpa* ~ 192 genes; *Linum usitatissimum* (flax) 167 genes (Yanhui et al. 2006), *Zea mais* (maize) 157 genes (Du et al. 2012), *Arabidopsis thaliana* 126 genes (Yanhui et al. 2006), *Oriza sativa* (rice) ~ 117 genes (Kang et al. 2022), and *Phalaenopsis aphrodite* 99 genes (Lu et al. 2022). These R2R3-MYB proteins are characterized by two distinct, adjacent R repeats (R2 and R3) (Fig. 1) and play key roles in regulating diverse physiological processes, such as stress response, metabolism, cell differentiation, morphogenesis, disease resistance, and seed development (Dubos et al. 2010; Yang et al. 2012; Ma And Constabel 2019; Pratyusha et al. 2022; Wu et al. 2022; Biswas et al. 2023; Ding et al. 2024; Mariyam et al. 2025; Zhang et al. 2025a). For more detailed information, see Table 1 in Ma et al. (2025).

**Table 1:** Representative GAMYB TFs and associated regulatory genes involved in seed germination, and reserve mobilization in agronomic crops

Gene	Function	References
HvGAMYB	Barley GAMYB interacts with the DOF TF BPBF to activate endosperm-specific genes, such as storage proteins, promoting endosperm maturation and nutrient accumulation. That is, HvGAMYB primes seeds for germination but doesn’t directly control it	Díaz et al. (2002)
HvMYBS3	Forms a ternary complex to coordinate gene activation for seed development and reserve mobilization	Rubio-Somoza et al. (2006)
HvVP1(Viviparous-1)	HvVP1, an ABA related TF, interacts with HvGAMYB to suppress hydrolase (e.g., α-amylase) gene activation, suggesting that VP1/ABA may repress GAMYB-mediated seed dormancy and germination. Namely, HvGAMYB acts in both maturation and germination, under regulatory control of HvVP1. HvGAMYB functional effects on germination are currently lacking	Abraham et al. (2016) Liew et al. (2020)
MYB33/MYB65	These TFs are post-transcriptionally regulated by the microRNA miR159. Both coordinate the transition from seed dormancy to germination in A. thaliana. LOF mutants alter sensitivity to GA. The myb33, myb65, myb101 mutants show reduced aleurone gene expression and impaired GA-mediated vacuolation during germination	Reyes and Chua (2007) Alonso-Peral et al. (2010)
LeGAMYBL1	Expressed in endosperm and embryo during seed germination and induced by GA, but not by ABA, indicating a role in promoting germination and early seedling growth	Gong and Bewley (2008)
GAMYB (review)	GAMYB (regulated by GA) activates hydrolase genes (i.e., α-amylase) in aleurone; but in wheat, its transcript levels do not always differ between dormant and after-ripened seeds, suggesting post-transcriptional or GAMYB-independent regulation	Gao and Ayele (2014)
TaGAMYB	Integrates ABA signaling to positively regulate seed dormancy and inhibit germination, acting as a key transcriptional regulator that links environmental cues with hormonal control of the dormancy–germination transition	Nguyen et al. (2025)
OsGAMYB	OsGAMYB, regulated by miR159, controls GA-responsive genes like α-amylase in the aleurone, promoting seed germination. Disruption of OsGAMYB or its regulation by miR159 impairs gene expression and germination efficiency	Tsuji et al. (2006) Millar et al. (2019)
ScGAMYB	ScGAMYB is highly conserved with wheat and barley orthologs, contains SNPs linked to α-amylase activity, and can be targeted with allele-specific markers for breeding. Still, variations in GAMYB do not consistently predict germination or dormancy	Bienias et al. (2020)
TaNAC019	While not directly linked to dormancy or germination, TaNAC019 regulates glutenin and starch by binding TaGlu-1 promoters, and its elite allele improves wheat grain quality	Gao et al. (2021)
GAMYB	During early rice seed germination, GA-responsive factors such as GAI and GAMYB activate genes involved in starch mobilization, coordinating both transcriptional and post-transcriptional regulation. GAI would transactivate GAMYB in vivo during germination. Data on functional GAMYB manipulation and its impact on germination percentage are lacking	Li et al. (2022b)
NtMYB330	NtMYB330 controls proanthocyanidin accumulation and seed germination in tobacco by forming an MBW complex. It acts as a key regulator of seed-coat traits and dormancy	Zhao et al. (2022)
TaGAMYB	By phosphorylating and stabilizing TaGAMyb, TaSG-D1 promotes TaCKX2.2.1 expression, decreases cytokinin levels, and negatively impacts grain development in wheat	Zhou et al. (2025)

A recent study identified 65 *R2R3-MYB* genes in the quinoa (*Chenopodium quinoa*) genome, which were classified into 26 subfamilies based on phylogenetic analysis (Ding et al. 2024). Expression profiling under saline–alkali stress conditions revealed that several of these genes are significantly regulated in response to abiotic stress. Furthermore, consistent with their homologs in *A. thaliana*, *CqMYB2R09*, *16*, *25*, and *62* showed transcriptional activation activity and nuclear localization, suggesting they play key roles in quinoa’s response to saline stress by modulating downstream stress-related genes. These findings indicate that the *R2R3-MYB* gene family in quinoa retains conserved molecular features found in other plant species while also exhibiting lineage-specific divergences that may contribute to adaptation to challenging environmental conditions (Ding et al. 2024).

The second group is the R1R2R3-MYB subfamily, which is similar to animal MYB proteins and contains three adjacent R repeats. The third group is a heterogeneous set collectively referred to as MYB-like proteins, which tipically—but not always—contain a single MYB repeat (Martin et al. 1997; Wu et al. 2024).

R2R3-MYB TFs which have been identified in over 70 land plant species (Wu et al. 2022, 2024), have been classified into 23–90 subgroups, though classification remains debated due to lineage-specific gene gains or losses and differences in sampling coverage, analytical methods, and subgroup criteria used across studies (e.g., comparative phylogenomic analyses revealing variable subfamily numbers and inconsistent subgroup nomenclature among taxonomic lineages) (Wu et al. 2022a; Xu et al. 2024).

## MYB TFs in plants: recent advances and functional insights (2015–present)

**Fig. 3:**
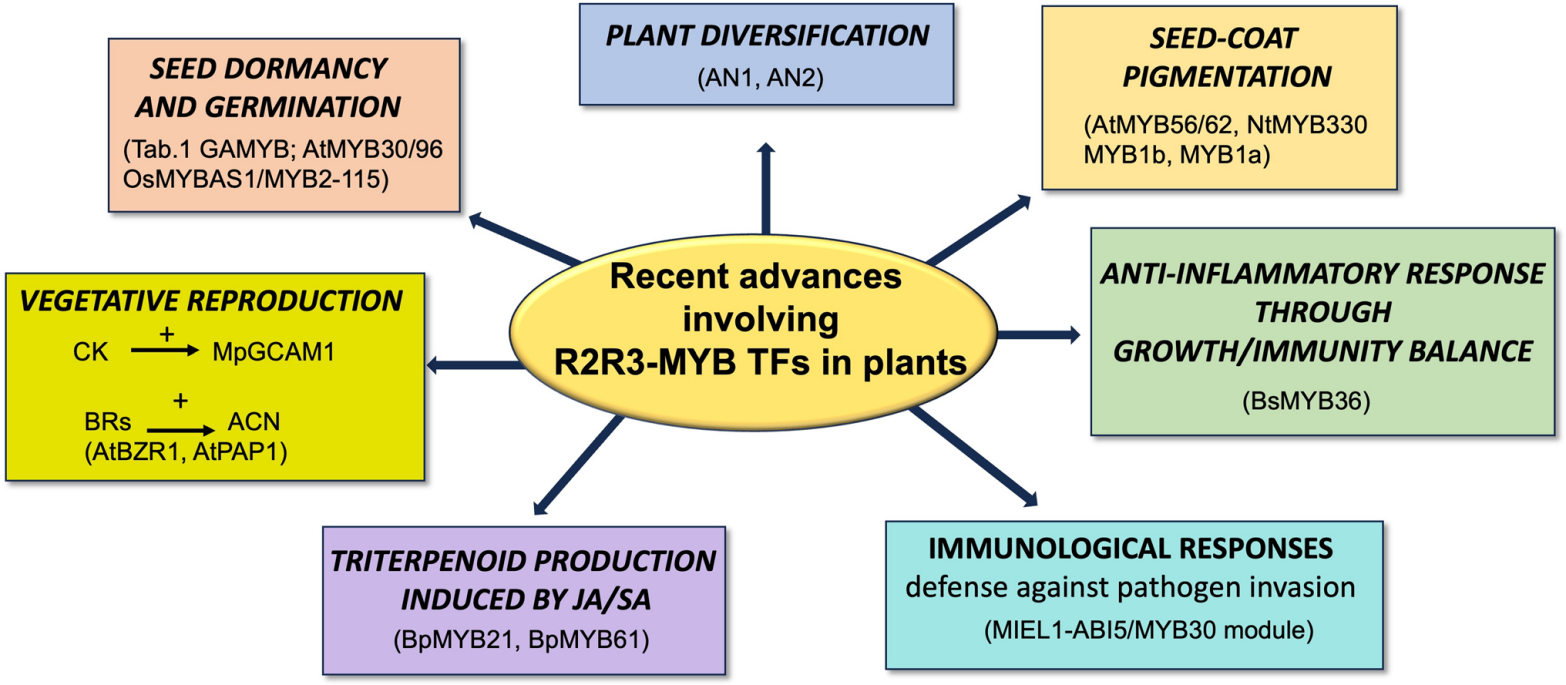
Involvement of selected R2R3-MYB TFs in key processes of the plant-life cycle. The figure summarizes some of the main roles these TFs play during critical stages of plant development, whose detailed description is provided throughout the text

MYB TFs, due to their abundance and functional diversity, are considered central regulators of plant-specific processes (Fig. 3). R2R3-MYB proteins are involved in a broad array of physiological and developmental pathways, including cell differentiation, organ development, stress responses, and secondary metabolism (Ma et al. 2019). Importantly, repressive MYB proteins are now recognized not simply as passive inhibitors, but as dynamic modulators that integrate multiple signaling pathways, thereby influencing the biosynthesis and allocation of phenylpropanoids—including FLAVs, ACNs, and lignin—within plants (Thakur et al. 2022).

In the context of secondary metabolism, manipulating MYB TFs—through overexpression or silencing—has emerged as a promising strategy to enhance the production of bioactive compounds in medicinal plants (discussed in more detail later in this section), with potential applications in bioprospecting, cultivation, and pharmaceutical applications (Thakur et al. 2022). Furthermore, recent studies on primary metabolism indicate that certain R2R3-MYB TFs also contribute to the regulation of lipid metabolism. For example, subgroup 1 MYB96 appears to promote triacylglycerol (TAG) biosynthesis by activating key enzymes, such as Acyl-CoA:diacylglycerol acyltransferase-1 (DGAT1) and phospholipid:diacylglycerol acyltransferase-1 (PDAT1), highlighting the versatile regulatory potential of these R2R3-MYB TFs (Lee et al. 2018). Interestingly, this study indicates that fatty acid biosynthesis and TAG accumulation are under independent transcriptional control, with MYB96 primarily responsible for TAG assembly in seeds.

### Vegetative reproduction

Some R2R3-MYB TFs act as lineage-specific regulators, while others retain conserved functions during evolution. Gemma cups are specialized structures that generate clonal propagules. Although carbon availability promotes gemma cup formation in *Marchantia nepalensis*, its mechanisms remain unclear; however, recent work in *Marchantia polymorpha* implicates cytokinin pathway activation and underscores the importance of carbon–cytokinin interactions in plant developmental plasticity (Humphreys et al. 2025). Notably, studies by Yasui et al. (2019) revealed that the R2R3-MYB GEMMA CUP-ASSOCIATED MYB1 (GCAM1), identified in *M. polymorpha*, one of the earliest-diverging lineages of land plants, is an ortholog of the *REGULATOR OF AXILLARY MERISTEMS* (*RAX*) genes in angiosperms. It functions as a master regulator of vegetative reproduction by controlling the formation of gemma cups. As a *RAX* ortholog, GCAM1 illustrates that MYB-mediated regulation of meristem development is a deeply conserved mechanism. This links early land plant development with broader MYB TF functions in organogenesis, secondary metabolism, and hormonal regulation in higher plants (Yasui et al. 2019).

Taken together, these findings suggest that the genetic program underlying meristem formation and vegetative propagation is deeply conserved across land plants. Recent studies revealed that the KARRIKIN INSENSITIVE-2 (KAI2)–dependent signaling pathway regulates vegetative reproduction in *M. polymorpha* by inducing MpLOG expression, a cytokinin biosynthetic enzyme, in a cell-type–specific manner. This activation increased cytokinin levels, revealing a regulatory cascade which subsequently induces the expression of *GCAM1,* revealing a regulatory cascade, KAI2 → LOG → Cytokinin → GCAM1, that controls vegetative reproduction in *M. polymorpha* (Komatsu et al. 2025). Moreover, Higo et al. (2018) demonstrated that the DUO POLLEN 1 (DUO1) TF, which arose through neo-functionalization, is essential for sperm cell differentiation in plants, regulating genes required for the formation, division, and functionality of male gametes. This work illustrates how the expansion and diversification of TF types can drive novel cellular functions and reproductive innovations during plant evolution (Higo et al. 2018). In more recent studies, the R2R3-MYB transcription factor MpSHOT GLASS (MpSTG) was identified as a key regulator of both sexual and vegetative reproduction in *M. polymorpha*, highlighting that conserved developmental mechanisms operate in bryophyte gametophytes and angiosperm sporophytes (Sakai et al. 2025).

### ACN biosynthesis

In 1987, the first MYB TF in plants, COLORED1 (C1) from *Zea mays*, which encodes the ZmMYBC1 protein, was isolated and identified (Martin and Paz-Ares 1997). This factor is involved in the regulation of ACN biosynthesis in corn pollen tissue (Paz-Ares et al. 1987). Years later, one of the most extensively studied MYB TFs is Production of ACN Pigment 1 (PAP1 or MYB75), known for its role in controlling ACN biosynthesis (Du et al. 2026; Huang et al. 2026). These FLAV pigments color plant tissues, attract seed dispersers, act as antioxidants, and provide defense against pathogens and herbivores.

In this context, Lee’s group demonstrated that in Arabidopsis seedlings, brassinosteroids (BRs) promote ACN accumulation by regulating PAP1 and PAP2 through BZR1, a key regulator of the BR signaling pathway (Lee et al. 2024). These findings highlight the complex crosstalk between MYB-mediated transcriptional control and hormone signaling in secondary metabolite production. BZR1 not only activates the expression of PAP1 and PAP2, but also interacts directly with them to cooperatively regulate genes involved in ACN biosynthesis, such as TT8 and LBGs. Likewise, under nitrogen-deficient conditions, the expression of BZR1 and PAP1 increases, enhancing their interaction and further promoting ACN accumulation. In summary, this recent study reveals that BZR1 acts as an integral component of hormonal regulation, linking BRs signaling with activation of the MYB–bHLH–WD40 (MBW) complex via PAP1 to promote ACN biosynthesis (Lee et al. 2024). Additionally, related work in *Solanum lycopersicum* highlights the broader role of BZR1 interacting proteins in BR dependent growth and stress responses, supporting the expanded functional network of BZR1 in plant development (Liu et al. 2026).

On the other hand, although APETALA2 (AP2) belongs to the AP2/ERF family rather than MYBs, it can negatively regulate proanthocyanidin (PA) biosynthesis by repressing MBW complexes, acting upstream of MYB TFs. In seeds, AP2 also modulates ABA/GA-related genes, affecting dormancy, germination, and embryo development—processes closely linked to NtMYB330 function (Jiang et al. 2024; Chen et al. 2026).

### Triterpenoids production

BpMYB21 and BpMYB61 were identified in birch (*Betula platyphylla*) by Yin et al. (2020) as two R2R3-MYB TFs that respond to salicylic (SA) and jasmonate (JA), two plant hormones involved in stress responses and secondary metabolism. These two TFs regulate triterpenoid biosynthesis with BpMYB21 acting as a strong activator, whereas BpMYB61 exhibits a more complex, possibly dual, regulatory role. The promoters of both genes are active, and their expression is tissue-specific and hormone-responsive, suggesting that they contribute to the fine-tuning of triterpenoid production (Yin et al. 2020). MYB TFs have similarly been implicated in triterpenoid biosynthesis in medicinal plant *Tripterygium wilfordii*, reflecting the evolutionary conservation of MYB-mediated regulation of secondary metabolites across plant species (Li et al. 2025).

In a related study, Zhang’s group, also working in *B. platiphylla*, showed that *BpMYB95* could serve as a valuable genetic resource for developing salt-tolerant birch varieties. Overexpression of this TF significantly enhances salt tolerance, reducing leaf chlorosis and wilting, and mitigating damage to photosystem II (PS II) under saline conditions (Zhang et al. 2024a). Recent work has expanded our understanding of R2R3-MYB TFs in *B. platyphylla*, with genome-wide identification of 111 R2R3-MYB genes and analysis of their stress-responsive expression profiles, including the functional characterization of BpMYB95 in salt tolerance (Zhang et al. 2025a, b, c, d).

### Plant immunity and stress responses

MYB TFs play central roles in plant immunity and stress responses at both transcriptional and physiological levels. They regulate multiple layers of defense by modulating defense-related genes expression hormone signaling pathways, and the biosynthesis of antimicrobial secondary metabolites, such as FLAVs, phenolics, and lignin. For instance, AtMYB30 enhances SA-dependent defense gene expression while antagonizing JA signaling, thereby balancing competing defense pathways. In addition, many MYB TFs also influence ethylene signaling, further fine-tuning the plant’s immune responses. That is, SA, JA and ethylene collectively orchestrate systemic acquired resistance (SAR) and induced systemic resistance (ISR), two essential mechanisms that enable plants to arrange effective and sustained immune responses. Beyond signaling, MYB TFs also reinforce structural barriers, such as the cuticle and cell wall, which constitute the first line of defense against pathogens (Tougeer et al. 2026).

Within this regulatory network, AtMYB30 has emerged as a multifunctional regulator integrating developmental and stress-related signaling networks. It acts as a molecular switch linking biotic (pathogen attack) and abiotic (environmental) stress responses, while also regulating lipid metabolism and plant development. At the post-translational level, AtMYB30 interacts with ABI5 and is targeted by the E3 ubiquitin ligase MIEL1, fine-tuning ABA-dependent seed germination and early seedling development (Nie et al. 2022). Moreover, nitric oxide (NO)-mediated S-nitrosylation at Cys-49 enhances its activation of the ABA catabolic gene *CYP707A2*, reducing ABA levels and promoting germination. This regulation is further modulated by the ABA receptor PYL4, whose inhibitory effect on AtMYB30 is relieved by S-nitrosylation, highlighting a tight interplay between hormonal and redox signaling (Zhao et al. 2024b). In addition, AtMYB30 interacts with the E3 ubiquitin ligases BTSL1 and BTSL2, protecting the bHLH transcription factor FIT from proteasomal degradation and thereby promoting iron-deficiency responses (Zhao et al. 2025a, b). Collectively, these findings demonstrate that AtMYB30 integrates ABA signaling, redox regulation, and protein stability control to ensure proper growth and stress adaptation in *Arabidopsis thaliana*.

On the other hand, the R2R3-MYB TF MdMYB30 has been shown to enhance disease resistance by directly modulating lipid metabolism and cuticular wax biosynthesis, thereby strengthening both the physical and chemical defense barriers against pathogens. Specifically, this TF activates the expression of the β-ketoacyl-CoA synthetase (*MdKCS1*) gene, which alters the composition of cuticular waxes and consequently increases resistance to pathogens, such as *Pseudomonas syringae* pv. *tomato* DC3000 and *Botryosphaeria dothidea* (Zhang et al. 2019).

Under drought stress, BpMYB123 serves as a central regulator that improves birch (*Betula platifilla*) drought tolerance by directly upregulating BpLEA14 (a LEA protein), thereby mitigating oxidative damage and protecting cellular membrane integrity during water deficit (Lv et al. 2021). A recent study has shown that PtrMYB94, an R2R3-MYB TF, is induced by drought and ABA and that overexpression of *PtrMYB94* can improve poplar drought tolerance (Fang et al. 2020). *Populus* plants overexpressing *PtrMYB94*, displayed increased tolerance to extreme drought stress via upregulation of *Embryogenic Cell Phosphoprotein-44* (*PtrECPP44*) expression (Kong et al. 2024).

Lastly, silencing or knocking down *OsMYB30* enhances cold tolerance in rice by promoting the expression of β-amylase genes, which boosts starch breakdown and maltose accumulation, thereby improving membrane protection under low-temperature stress. Moreover, OsMYB30 interacts with the OsJAZ9 protein, which enhances its repressive effect on β-amylase genes. This complex contributes to modulating the plant’s response to cold stress (Gu et al. 2021; Zhao et al. 2024c).

In summary, R2R3-MYB TFs, such as AtMYB30 (*Arabidopsis thaliana*), MdMYB30 (*Malus domestica*), and OsMYB30 (*Oryza sativa*), share key features: (i) regulation of defense-related genes; (ii) involvement in oxidative stress, JA signaling, and cutin biosynthesis; and (iii) conservation of functional motifs associated to abiotic and biotic stress responses. Overall, MYB TFs act as central regulators that finely balance growth and immunity, enhancing plant resilience to diverse environmental challenges (Bowman et al. 2017; Stracke et al. 2001; Kang et al. 2022; Wu et al. 2022a).

As stated above, R2R3-MYB TFs play essential roles in regulating the biosynthesis of FLAVs secondary metabolites (Mao et al. 2025). In this regard, FLAVs are major constituents of *Bletilla striata* (commonly known as Chinese ground orchid, characterized by its pinkish-purple flowers and valued as a medicinal plant), and contribute to its anti-inflammatory and antioxidant properties (Ma et al. 2021). Considering the reported biological properties of *B. striata*, a comprehensive investigation of R2R3-MYB TFs family was carried out, leading to the identification of 94 MYB genes. Among them, *BsMYB36* and *BsMYB51* were identified are key R2R3-MYB TFs that critically regulate FLAV accumulation in this plant, underscoring their central role in the metabolic and regulatory networks of orchids and shedding light on the functional evolution of the R2R3-MYB gene family. The absence of signal peptides in their proteins indicates that both BsMYB36 and BsMYB51 were non-secretory proteins (Huang et al. 2025).

Notably, functional assays demonstrated that *BsMYB36* (S7) and *BsMYB51* (S5) could complement *A*. *thaliana* mutants (*atmyb123* and *atmyb12*, respectively). Furthermore, overexpression of BsMYB36 increased FLAV levels while reducing ACN and proanthocyanidin accumulation, whereas BsMYB51 enhanced the accumulation of all three compounds. *BsMYB36* and *BsMYB51* upregulated key enzymes in the phenylpropanoid and FLAV biosynthetic pathways (e.g., PAL, CHS, F3’H, DFR). These findings provide a basis for understanding BsMYB-mediated regulation of FLAV metabolism in *B. striata* (Huang et al. 2025). Taken together, MYB-regulated FLAVs play a key role in plant defense against biotic and abiotic stresses and, as secondary metabolites, can also exhibit pharmacological properties, such as anti-inflammatory and antioxidant activities (Liu et al. 2015; Rabeh et al. 2025; Shen et al. 2019).

To conclude this section on immunity, JA is essential for antiviral defense, as viral infection triggers JAZ protein degradation, releasing TFs to activate JA-dependent defense genes (Zhao et al. 2024c). OsMYB4P, a JA-responsive R2R3-type MYB TF in rice, activates JA biosynthesis and enhances antiviral defense. It is regulated by OsJAZs and disrupted by viral proteins SP8 (SRBSDV) and P2 (RSV), which reduce its stability and activity, suppressing JA signaling. Thus, OsMYB4P is a key JAZ-targeted regulator of JA-mediated antiviral immunity (Lu et al. 2025). Overall, this group identifies OsMYB4P as a novel JA‐responsive R2R3‐type MYB TF that acts as a direct target of OsJAZs to specifically mediate JA‐regulated broad‐spectrum antiviral immunity in rice. Summarizing, JA → JAZ degradation → OsMYB4P activation → immune defense.

### Diversification in plants

The R2R3-MYB TF family is one of the largest and most diverse regulatory systems in plants, expanded mainly through whole-genome and small-scale duplications (Jiang And Rao 2020; D’Amelia et al. 2025). This expansion has generated numerous conserved and lineage-specific subfamilies, reflecting a highly dynamic and uneven evolutionary diversification among clades (Jiang And Rao 2020; Wu et al. 2022). Genomic analyses indicate that R2R3-MYB complements often exceed one hundred members organized into multiple subgroups (D’Amelia et al. 2025). In Solanaceae, Gates et al. (2016) investigated the evolution and diversification process of the R2R3-MYB gene family identified 559 members across four species and reconstructed their phylogeny, revealing a major (~ 54%) expansion in the ancestral angiosperm lineage followed by lineage-specific gene gains and losses maintained over long evolutionary timescales, supporting the emergence of novel, lineage-specific functions.

Their functional diversification is driven by changes in regulatory regions and protein architecture: while the R2R domain is highly conserved, the C-terminal region shows substantial variability in functional motifs (Jiang And Rao 2020). In Solanaceae, R2R3-MYB genes have followed highly dynamic evolutionary trajectories involving duplication and functional divergence, yet the overall size of the family has remained relatively stable for ~ 90 million years (Gates et al. 2016). Recent work by D’Amelia et al. (2025) suggests that asymmetric duplications—particularly in subfamily VIII—have driven diversification, with high gene retention indicating acquisition of new functions under selective pressure.

Additionally, R2R3-MYB proteins function as pleiotropic regulatory nodes within complex networks, coordinating processes, such as stress responses and metabolism (D’Amelia et al. 2025). Overall, their diversification arises from the interplay of gene expansion, regulatory sequence evolution, and network reorganization, rather than a single dominant mechanism (Jiang And Rao 2020; D’Amelia et al. 2025). This integrative framework explains their functional versatility and their central role in plant regulatory complexity and lineage-specific adaptation.

### R2R3-MYB TFs and phytohormones

R2R3-MYB TFs are tightly connected to plant hormone metabolism (i.e., CKs, JA, BRs and SA), integrating hormonal signals with developmental and stress-responsive pathways (Fig. 3). In recent years, notable advances have deepened our understanding of how R2R3-MYB TFs interact with major phytohormones, such as GAs, IAA, ethylene and ABA. Thus some R2R3-MYB TFs act downstream of GAs to regulate processes, such as cell elongation, flowering, and secondary metabolism, while other family members influence GAs biosynthesis and breakdown—forming a regulatory feedback loop that refines GAs-driven development.

Recent studies in the early 2020 s have highlighted the central roles of R2R3-MYB TFs in plant growth, development, and stress responses. For instance, OsMYB14 in rice exemplifies how these TFs integrate multiple hormonal pathways, as it regulates plant height by modulating both GAs and auxin metabolism (Kim et al. 2024). Meanwhile, PtoMYB142 promotes drought tolerance in *Populus tomentosa* by enhancing GAs catabolism and optimizing leaf structure (Song et al. 2024). Similarly, in Brassica rapa, BrMYB108 extends leaf lifespan by influencing auxin-regulated leaf senescence (Liu et al. 2025), whereas AtDIV1 restricts root growth in Arabidopsis through PIN5-mediated auxin transport (Dai et al. 2025). However, despite these diverse roles in growth regulation, R2R3-MYB TFs have not been shown to directly regulate ethylene biosynthesis or signaling, ethylene-driven pathways can activate repressor-type MYBs to mediate physiological effects (Wang et al. 2024a, b). Finally, PsMYB306 is induced by exogenous ABA and positively correlates with the ABA biosynthetic gene *PsNCED3*, linking MYB activity to stress-responsive hormonal regulation (Yuan et al. 2024).

On the other hand, the *GhODO1* (*Gossypium hirsutum* ODORANT1) gene was recently identified (Zhu et al. 2022). This gene enhances resistance to *Verticillium dahliae* (the causal agent of Verticillium wilt) by activating lignin biosynthesis genes (e.g., *Gh4CL1* and *GhCAD3*) through its binding to their promoters, thereby causing lignin accumulation. The involvement of JA signalling in this physiological process suggests that GhODO1 may also influence or integrate hormone-signalling responses in defence, and not only structural (lignin-based) responses. Conversely, *GhODO1* knockdown impaired JA-mediated defence signalling and decreased JA levels (Zhu et al. 2022).

Collectively, these recent studies demonstrate that R2R3-MYB TFs function as central integrative regulators. They coordinate phytohormone signalling with structural, physiological, and metabolic defence pathways, thereby contributing to the fine-tuning of plant stress-response networks. Nevertheless, while progress has been made unravelling this complex phytohormone–R2R3-MYB regulatory landscape, further research is required to fully understand it not only in model systems, but also in species of agronomic importance.

## Roles of R2R3-MYB TFs in zygotic embryogenesis and the regulation of seed dormancy and germination

As reflected in the above paragraphs of this text and summarized in Fig. 3, R2R3-MYB TFs are widespread in plants because they perform a wide range of vital functions throughout their life cycle. Currently, knowledge of these TFs is still insufficient, both in model systems (i.e., Arabidopsis, rice and soybean) and across diverse species, restricting our understanding of their genetic conservation. Dormancy and germination of seeds are two highly coordinated physiological processes governed by sophisticated molecular networks (Matilla 2025). Despite this, only a few studies have explored the role of R2R3-MYB TFs in these processes, leaving significant gaps in our understanding of their function during key stages of SLC. One possible explanation for this may lie in the complexity of the networks that regulate dormancy and germination.

This section highlights recent findings on the role of R2R3-MYB proteins in seed dormancy and germination, the underlying molecular mechanisms, and future directions for research and biotechnological applications. Specifically, we examine a number of representative R2R3-MYB TFs that contribute to the physiological, metabolic, and hormonal environments influencing seed dormancy and germination. Although each of them plays a role in shaping dormancy-related traits, none has been identified as the direct molecular switch that initiates the dormancy program. Instead, their functions appear to be predominantly indirect, preparatory, or modulatory—affecting ABA dynamics, seed-coat properties, or maturation processes—rather than directly triggering dormancy or germination.

### R2R3-MYB TFs and seed-coat pigmentation

AtMYB56/GmMYB62: in the stages leading up to the induction of seed dormancy, members of the R2R3-MYB TFs family have been reported to play critical roles. In 2015, Chen’s group identified the R2R3-MYB56 TF, an unstable protein that is degraded by the CRL3–BPM E3 ligase complex, which targets specific proteins for proteasome-mediated degradation. This post-translational regulatory mechanism allows Arabidopsis to fine-tune the biological processes controlled by this TF, including early seed development, pigmentation regulation, hormonal regulation, and various environmental responses (Chen et al. 2015).

Furthermore, functional analyses show that *AtMYB56*, a TF belonging to the S21 subfamily of MYB family (Dubos et al. 2010), is predominantly expressed in early developing seeds, where it promotes outer integument development in the seed coat by increasing cell number, thereby regulating seed size. It also controls cell wall genes essential for cell division and expansion (Zhang et al. 2013). Moreover, MYB56 acts maternally, and *myb56* mutant seeds are smaller than those of the wild type (WT); this function appears to be evolutionarily conserved (Zhang et al. 2013). Recent research has revealed that hormonal signals, particularly JA, influence seed size via additional genes beyond MYB56 and through mechanisms affecting protein stability, underscoring the multifactorial nature of seed-size regulation (Zhang et al. 2025a).

Similarly, GmMYB62 is a soybean R2R3-MYB TF that represses ACN biosynthesis by modulating the expression of key biosynthetic genes. In vivo, it acts as a key regulator coordinating seed color and size: it alters pigmentation by repressing ACN biosynthesis in the seed coat, while simultaneously controlling cell expansion and proliferation in the embryo and seed coat, thereby determining seed size and weight in Arabidopsis. These findings indicate that GmMYB62 functions as a dual regulator of pigmentation and seed development (Zhao et al. 2025b).

Interestingly, in pear (*Pyrus bretschneideri*), the combined overexpression of *PbMYB56* and *PbDELLA* (i.e., DELLA proteins play a key role as negative regulators in GA signaling) led to reduced seed development and loss of parthenocarpy potential in tomato (Gómez et al. 2023; Zhang et al. 2025c). These findings indicate that MYB56 function in seed development is tightly regulated—and in some cases suppressed—by the GAs signaling pathway via DELLA proteins.

On the other hand, Han’s group showed that BnaCO_3._MYB56 binds directly to the promotor of *BnaA09.LEC1*, a key gene involved in seed development, thereby activating its expression. Therefore, MYB56 can be considered a direct and positive regulator of the LAFL network (LEC1–ABI3–FUS3–LEC2), which plays a central role in embryogenesis and seed maturation (Pelletier et al. 2017; Han 2025).

NtMYB330: many R2R3-MYB TFs that specifically regulate PAs have been studied (Dixon et al. 2020). In herbaceous species, particularly in legumes, such as *Medicago*, both PAs and ACNs originate from the same metabolic pathway, are deposited in the seed coat and contribute to intense pigmentation and increased seed-coat thickness. They also play a key role in maintaining seed dormancy and inhibiting germination. This effect is likely mediated through their influence on ABA biosynthesis and/or by reducing seed-coat permeability to water (Wang et al. 2025b; Wen et al. 2025).

Recent results from Zhao et al. (2022) showed that NtMYB330: (i) specifically controls PAs biosynthesis in tobacco flowers and seeds. Mutation of *ntmyb330* promoted seed germination, whereas the overexpression of *NtMYB330* inhibited seed germination under normal conditions; and (ii) regulates the expressions of *NtNCED1*, *NtGA20ox2*, and *NtABI3*. In summary, this study identifies NtMYB330 as a PA-specific MYB regulator in tobacco that controls PAs biosynthesis via an MBW complex and affects seed germination by modulating PAs levels and ABA/GA signaling in seeds.

IbMYB1a: In *Nicotiana tabacum*, expression of *IbMYB1a* under different promoters produces distinct tissue-specific patterns and levels of ACN accumulation, showing that IbMYB1a positively regulates multiple ACN biosynthetic genes and highlighting its practical potential for crop pigmentation (Chu et al. 2013). By contrast, although the R2R3-MYB TF IbMYB1a has no known in vivo function in seeds, heterologous overexpression in soybean is sufficient to trigger the ACN biosynthetic pathway leading to pronounced ACN pigmentation in the cotyledons (An et al. 2015). In summary, An et al. (2015) were the first to demonstrate that ACN accumulation in soybean cotyledons can be achieved via transformation of a single gene (i.e., a transgenic approach). A 2024 study replicated and extended this approach using IbMYB1a, confirming that overexpression of a single MYB effectively activates ACN in cotyledons and underscores its potential for improving crop pigmentation (Yeom et al. 2024).

Recently, Yin’s group comprehensively mapped R2R3-MYB genes in colored rice, revealing structural and functional diversification as well as conserved evolutionary clades. They also identified tissue-specific regulatory patterns and key members involved in ACNs biosynthesis, highlighting their significance in transcriptional regulation and potential for crop improvement (Yin et al. 2024).

### R2R3-MYB TFs and seed dormancy and germination

AtMYB96: is one of the best-characterized R2R3-MYB TFs linked to seed dormancy. Its expression is strongly induced by ABA in seeds, but not by GAs. AtMYB96 mediates a variety of ABA-dependent plant responses, including ACNs accumulation, which occurs during the late stages of zygotic embryogenesis. It promotes primary dormancy by enhancing ABA biosynthesis through the activation of genes, such as *NCED2* and *NCED6*, while simultaneously repressing germination by inducing *ABI4*, which acts epistatically to MYB96. The MYB96-*ABI4* module also specifically regulates lipid mobilization in the embryo (Lee et al. 2015a, b).

In the same way, Lee et al. (2015c) conclude that: (i) MYB96 is an important regulator that fine-tunes primary seed dormancy by coordinating ABA and GA metabolism and does not act as the sole initiator of dormancy, but it significantly modulates the hormonal balance that determines the depth of primary dormancy; (ii) E3 ligase MIEL1 regulates the stability of MYB96 (Lee And Seo 2019); and (iii) MYB96-HDA15 (histone deacetylase) complex epigenetically silences ABA repressors, boosting ABA responses (Lee And Seo 2019).

In summary, MYB96 functions as a central regulatory node that integrates multiple molecular pathways to coordinate seed dormancy and germination, primarily by modulating ABA biosynthesis and controlling lipid reserve mobilization.

Most recently, *BoMYB96* and *BoMYB2* were identified in *Brassica oleracea* as TFs that bind the *BoABI5* promoter, repressing its transcription. Overexpression of these genes in *A. thaliana* and *B. napus* resulted in ABA insensitivity and enhanced germination under ABA treatment. These findings demonstrate the functional conservation of AtMYB96 and AtMYB2 in ABA signaling and provide new insights into ABI5 regulation in *B. oleracea* and *B. napus* (Shen et al. 2026).

AtMYB30: Nitric oxide (NO) antagonizes ABA-mediated inhibition of seed germination and seedling growth by the S-nitrosylation of ABI5 (Albertos et al. 2015), and the R2R3-MYB30 TF acts as a negative regulator of ABA signaling, as evidenced by reduced ABA sensitivity in MYB30-overexpressing seeds and its ability to repress ABA-responsive gene expression either by direct promoter binding or by inhibiting ABI5 activity (Zhen et al. 2012; Nie et al. 2022). However, the function of MYB30 in seed dormancy and germination is still largely unknown.

More recently, MYB30 has been shown to participate in NO-induced seed dormancy breaking via its own S-nitrosylation at multiple residues, fine-tuning ABA signaling and promoting germination by directly activating ABA catabolism through binding to the *CYP707A2* promoter, collectively indicating that AtMYB30 integrates NO and ABA pathways and primarily functions in promoting seed germination rather than in establishing dormancy (Zhao et al. 2024a; 2025b).

OsMYBAS1, OsMYB2-115 and GAMYB in modern agriculture: R2R3-MYB TFs are now widely recognized for their contributions to enhancing seed germination, vigor, and overall performance, making some of them as promising targets for modern agriculture. For example, the rice gene *OsMYBAS1* was characterized in 2022 as a positive regulator of germination under unfavorable conditions, acting through modulation of cellular antioxidant capacity (e.g., maintaining membrane integrity). Thus, at a burial depth of approximately 4 cm, *OsMYBAS1*-overexpressing plants exhibit higher antioxidant enzyme activity, improved germination, longer roots, and taller seedlings compared to WT wild type plants, whereas *OsMYBAS1* knock-out mutants show reduced germination (Wang et al. 2022).

On the other hand, under aging-simulating conditions, *OsMYBAS1*-overexpressing lines show significantly higher germination rates than both the knock-out mutants and WT plants (Wu et al. 2022b; Thianshum et al. 2024). For these reasons, OsMYBAS1 is emerging as a key genetic target in rice breeding, with potential to enhance seed quality, boost stress tolerance, and support more efficient agricultural practices (Fu et al. 2025).

Further exploring the role of R2R3-MYB TFs in modern agriculture, in 2024, Zhang’s group selected the gene *OsMYB2-115* from the 117 R2R3-MYB members identified in rice. This TF shows strong induction under salt stress across several vegetative and reproductive organs, indicating a systemic response. However, its expression is not detected in seeds (Zhang et al. 2024b). Nevertheless, *OsMYB2-115* represents a promising genetic candidate for developing rice varieties with enhanced salt tolerance—an increasingly relevant trait for large agricultural regions affected by soil salinization. Generating plants with altered *OsMYB2-115* expression, which are not yet available, will open a valuable line of investigation with strong potential for agronomic application.

Although both studies (i.e., Yang et al. 2022 and Zhang et al. 2024b) employ similar methodologies, the former provides a general characterization of the R2R3-MYB TF family in tobacco, whereas the latter examines this family in rice with a specific functional focus on salt stress responses.

In 1995, Gubler and colleagues initiated the functional characterization of R2R3-TFs of the GAMYB in cereals (i.e., wheat and barley) as activators of GA-regulated genes in aleurone layer of *Hordeum vulgare* seeds, particularly during GAs-regulated germination (Gubler et al. 1999). Five years later, the same group demonstrated that GA signaling in barley aleurone cells regulates seed germination by promoting GAMYB expression and repressing the DELLA protein SLN1, thereby activating GAs-dependent genes required for reserve mobilization (Gubler et al. 2002).

Isolation of GAMYB homologs in several cereal species subsequently showed that the GAs-dependent activation mechanism of α-amylase is conserved across grasses (Haseneyer et al. 2008). Recent reviews on rice germination indicate that GAMYB/MYBGA binds to conserved *cis*-elements in the α-amylase promoter and activates its expression in the aleurone layer, thereby playing an essential role in the induction of this enzyme (Damaris et al. 2019). Basal land plants, including mosses, possess a GAMYB-based system that regulates spore and sexual organ development, indicating a deeply conserved ancestral function (Aya et al. 2011).

The function of GAMYB in modern agriculture has been investigated primarily through representative TFs involved in seed dormancy release and germination, including barley HvGAMYB, wheat TaGAMYB, and the Arabidopsis GAMYB-like factors MYB33 and MYB65 (Table 1). In agronomic species, these roles are exemplified by orthologs, such as rye *ScGAMYB*, supporting a conserved regulatory function of GAMYB across plant lineages. Nevertheless, the contribution of GAMYB to seed germination in major crops, including maize, remains to be elucidated.

### TT1 first, TT2 (R2R3-MYB TF) follows: orchestrating seed-coat development, dormancy and germination

The co-regulator TRANSPARENT TESTA 1 (TT1) is a C2H2-type zinc finger TF that was identified in young seeds of *Arabidopsis thaliana* through reverse genetics by the Sagasser’s group. Mutation of *TT1* (*tt1*) produces seeds with a transparent, (yellowish) and permeable testa due to a severe deficiency in FLAV accumulation, specifically a lack of PAs in the seed endothelium (Sagasser et al. 2002)*.* Interestingly, BnA09MYB47a, an R2R3-MYB TF unrelated to TT1, controls seed-coat pigmentation and FLAV biosynthesis in *Brassica napus*. It is a key regulator of seed-coat color, essential for PAs and ACNs biosynthesis, and a promising target for breeding high-quality seeds (Qu et al. 2023).

**Fig. 4:**
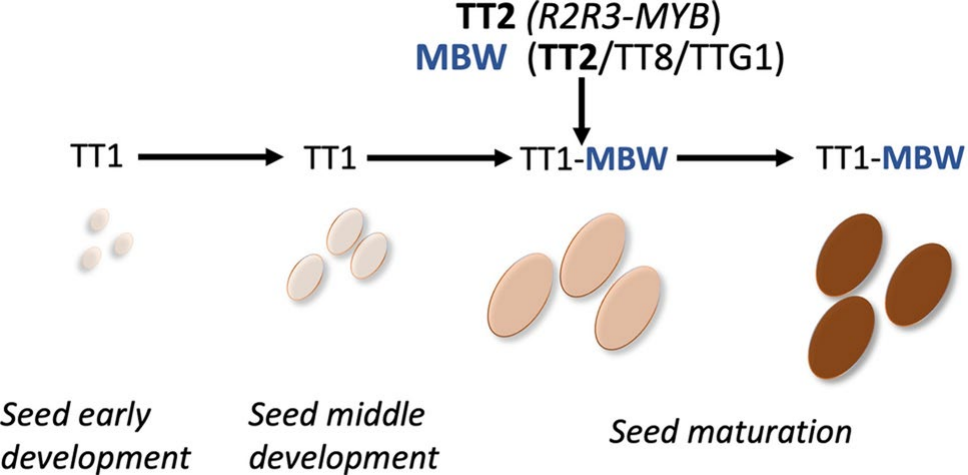
Temporal dynamics of TT1 (early regulator) and TT2 (late regulator) across early, middle, and maturation stages of *Arabidopsis thaliana* zygotic embryogenesis. TT1 functions during the initial stages of seed formation. It is required for proper endothelium differentiation and establishes the cellular framework necessary for subsequent regulatory events, thereby serving as a prerequisite for TT2 activity. In contrast, TT2 acts only after the seed-coat tissues have been specified by TT1 and activates the expression of late biosynthetic genes involved in PAs production, which ultimately determine seed-coat pigmentation. Regarding seed dormancy, TT1 influences this trait primarily through structural modifications of the seed coat: *tt1* mutants exhibit increased permeability and consequently show an early loss of dormancy. TT2, by contrast, contributes to dormancy mainly by modulating PA levels: *tt2* seeds lack PAs and show a mildly reduced dormancy phenotype. Importantly, TT2 cannot compensate for the absence of TT1; without TT1, TT2 is unable to activate PA biosynthesis because the target tissue fails to differentiate properly. Ultimately, TT2 does not act alone: requires partners to function. In the MBW complex, a master regulatory module that controls late FLAVs gene expression in developing seed coat, TT2, TT8, and TTG1 provide DNA-binding specificity, transcriptional activation, and structural stabilization of the complex, respectively

All *tt* mutant seeds display pigmentation and structural defects in the seed coat. These maternally inherited alterations are consistently associated with increased seed permeability, which corresponds to lower dormancy. The biochemical basis of this increased permeability is not yet fully understood. *TT1* is implicated in the regulation of genes involved in both the early stages of FLAV biosynthesis, including *Chalcone Synthase,* (*CHS*)*;* and *Chalcone Isomerase* (*CHI*)*,* and the late stages, including *Anthocyanidin Reductase* (*ANR*) and *Anthocyanidin Synthase* (*ANS*); these processes occur in the endothelium, the inner layer of the seed coat.Yeast two-hybrid (Y2H) assays demonstrated that TT1 physically interacts with the R2R3-MYB TF *TT2* (Fig. 4). Through this interaction, TT1 functionally contributes to the TT2/TT8(bHLH)/TTG1(WD40) regulon (the MBW protein complex) and acts (Appelhagen et al. 2011*).* TT2 is essential for the accumulation of PAs in developing seeds, and *tt2* mutants lack PAs, confirming that TT2 is a key determinant in this process (Nesi et al. 2001*)*.

In a comprehensive study, Coen et al. (2020) showed that the endothelium of the seed coat displays position-dependent characteristics that reflect its intrinsic polarity. TT1 contributes to establishing this polarity, whereas *tt1* mutants exhibit altered cell orientation. Accordingly, TT1 is required for proper endothelium architecture and regulates a gene network that is distinct from the one controlling pigment biosynthesis. Thus, TT1 functions not only in pigmentation, but also in coordinating the structural organization of the seed coat (Coen et al. 2020). These pronounced functional differences between TT1 and *tt1* may underlie the observed alterations in seed dormancy and germination, although fully elucidating this complex scenario will require extensive further study.

With respect to the aim of this review, no evidence indicates that TT1 directly regulates classical hormonal regulators of seed dormancy, such as ABA, DOG1, ABI3, ABI5; nevertheless, TT1 can influence dormancy indirectly through its role in seed-coat development and consequently *tt1* mutants display reduced dormancy and increased germination capacity.

Notably, the Lian’s group recently cloned and silenced *BnTT1* genes in *B. napus* using RNA interference (RNAi). The results showed that suppression of *BnTT1* genes not only reduced FLV accumulation, but also significantly caused abnormal testa development (Lian et al. 2017). However, this study provides no evidence that *BnTT1* silencing affects seed dormancy or germination. Related to this work, the Fan’s group generated targeted mutants of six key FLAV biosynthetic pathway genes—three structural (*BnaTT7*, *BnaTT8*, *BnaTT10*), two regulatory (*BnaTT1*, *BnaTT2*), and one transporter (*BnaTT12*)—in *B. napus* (Fan et al. 2023/24). These six mutants altered seed-coat color, modified fatty acid composition, and increased oil content without affecting germination, revealing functional divergence from *Arabidopsis* and a more complex regulatory network in this allotetraploid species. Recently, targeted mutation of *BnTTG1* using CRISPR/Cas9 accelerated seed germination under salt and cold stress (Cheng et al. 2024).

Homozygous knockout mutants exhibited diverse seed-coat colors, indicating functional divergence of these *BnaTT*s from Arabidopsis and a more complex flavonoid regulatory network in allotetraploid rapeseed. The yellow seed phenotype was most pronounced in the *Bnatt10* mutant (pure yellow), whereas *Bntt1* ranked sixth. Previous studies have shown that yellow seeds, which are considered as a high-quality trait, are generally associated with high oil content in both Arabidopsis and rapeseed (Zhang et al. 2022). In *B*. *napus*, *BnaTT8* and *BnaTT2* are essential for both seed coat color and oil accumulation. The six mutants studied showed no major germination defects, consistent with reports that most yellow-seeded oilseeds are morphologically normal (Lian et al. 2017; Zhai et al. 2020).

The results suggest that FLAV deficiency in the seed coat may influence oil content in *Bnatt* mutants. Notably, this study used targeted mutagenesis of FLAV biosynthesis genes to uncover key determinants of seed-coat color and offers germplasm for developing yellow‐seeded rapeseed. In this context, given the large R2R3-MYB family, the intrinsically disordered protein MYB73 was identified after the *A*. *thaliana* genome was sequenced. Jia et al. (2011) carried out its first functional analysis. More recently, MYB73 was shown to repress seed-oil biosynthesis and to contribute to seed-coat pigment biosynthesis (Yang et al. 2025). However, its functions differ markedly from those of TT1/TT2, to the extent that Yang’s group does not include it among their target genes.

In recent years, a hydrophobic, lipid-rich thick cuticle (CU) surrounding the endosperm has emerged as both a barrier preventing organ fusion and a key maternally derived structure regulating dormancy, germination, and seed viability (Matilla 2025). All studied tt mutant seeds exhibit severe defects in CU structure, increased permeability, and reduced dormancy, although the precise changes in the CU during normal germination remain unclear, and no CU mutant shows significant defects in radicle elongation under standard conditions. The CU also mediates endospermic ABA uptake, and endosperm TPST-sulfated CIF2 and PSY1 peptides promote seedling cuticle formation, highlighting its role in coordinating germination processes (De Giorgi et al. 2021; Pankaj et al. 2025).

In summary, TT1, although not directly involved in CU biosynthesis, does affect its deposition onto the endosperm. When TT1 is mutated or silenced, the synthesis of FLVs and PAs is altered, and the CU becomes thinner and more permeable. That is, the maternal gene TT1 affects CU quality, making it more defective (Loubéry et al. 2018).

## Phylogenetic origin and diversification of seed R2R3-MYBs

The phylogenetic analyses of plant R2R3-MYB transcription factors reveal an extraordinary diversification of genes grouped into well-supported clades or subfamilies (Bowman et al. 2017; Wilkins et al. 2009). These structural and sequence variations are framed by conserved regions within the R2R3 DNA-binding domain and diagnostic motifs in the C-terminal regulatory region (Fig. 1). Several subdivision proposals (Dubos et al. 2010; Li et al. 2012; Soler et al. 2015; Du et al. 2015; Bowman et al. 2017), some of them not though not completely congruent, have attempted to cover the full range of sequence diversity in R2R3-MYB. Here, we adopt the recent approach of Jiang and Rao (2020), which provides the most comprehensive phylogenetic study of R2R3-MYB genes, encompassing gene sequences across all groups of terrestrial as well as algal plants.

A selection of seed R2R3-MYB TFs described in the preceding sections were systematically retrieved and compared. To evaluate the extent of functional conservation and their assignment to defined phylogenetic subfamilies, based on amino acid sequence homology and gene structural features, we conducted a comparative maximum-likelihood phylogenetic analysis of the gene set reported in Table 1.

**Fig. 5:**
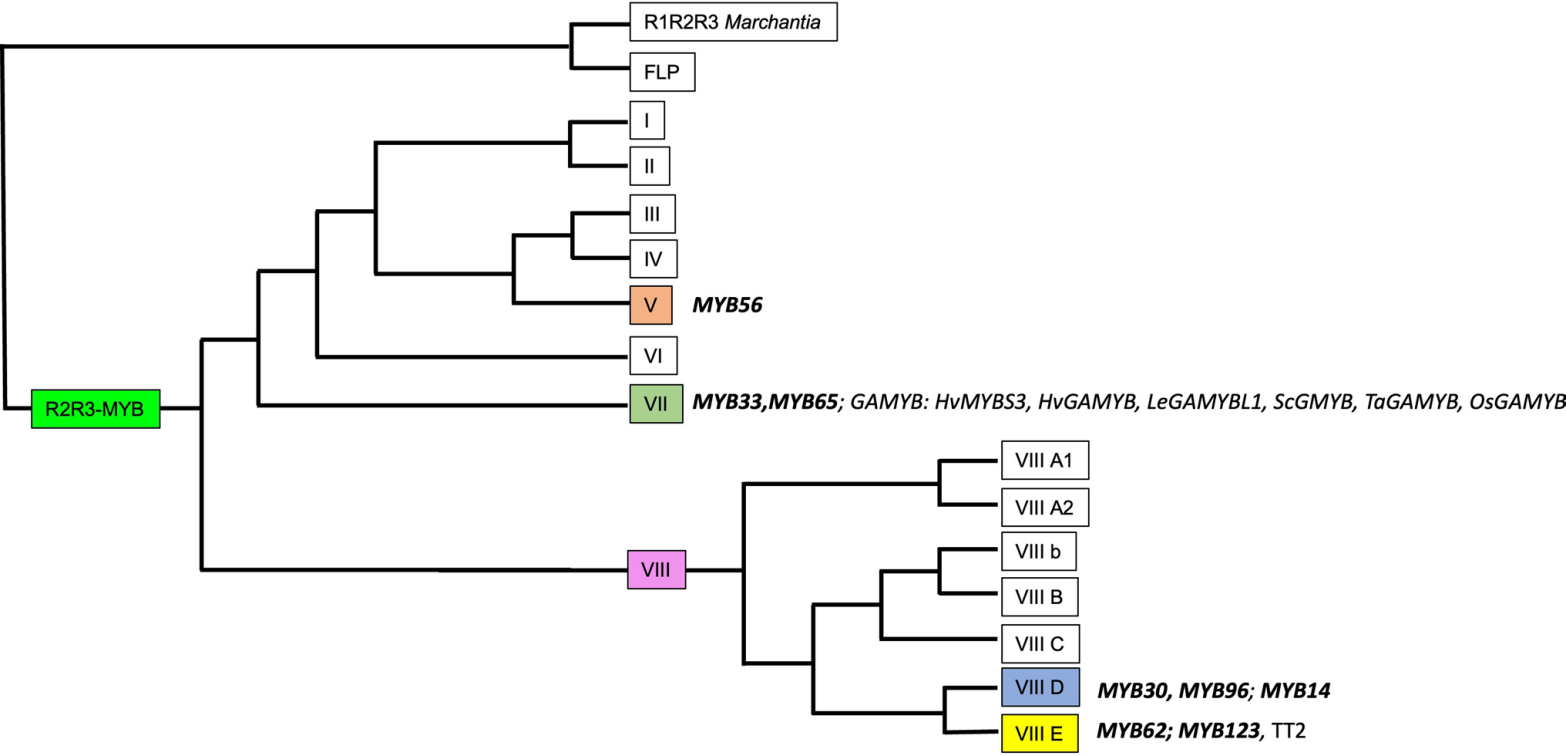
R2R3-MYB subfamily and clade ascription for genes mentioned in this review, based on Maximum -likelihood analyses of protein gene structure and protein aminoacid sequence. Phylogenetic arrangement is adapted from Jiang and Rao (2020). Bold names refer to *Arabidopsis thaliana* orthologs

Our study showed that all the R2R3-MYB genes involved in the regulation of seed dormancy and germination treated in this review belong to 3 subfamilies: V, VII and VIII (Fig. 5). The first group of genes include AtMYB56 and their orthologs, which belong to the subfamily V, also known as S21 subfamily in Dubos et al. (2010) classification. This group of TFs is part of a clade already present in Charophyceae genomes, and appears as a member of a lineage diversified after the split of Mesostigmatophyceae-Chlorokybophyceae. Its role as a regulator in seed pigmentation is conserved across Angiosperms, but its function in earlier diverging lineages is still not clear (Jiang And Rao 2020). The second group of genes, most of them informally recognized as GAMYB genes, belong to the subfamily VII of R2R3 MYB TFs. This subfamily, which roughly corresponds to Dubos subclass 18, constitute a distinct, evolutionarily conserved clade that specialize in hormone signaling and stress-responsive regulation. The orthologs in this subfamily appear in plant evolution as early as in Coleochaetophyceae, a subfamily of Charophytes (Jiang And Rao 2020) and are present prior to terrestrialization and seed plant origin. In angiosperms, although initially discovered from cultivated species by its critical regulatory functions, they can be phylogenetically defined by their common ancestry with AtMYB33 and AtMYB65. This monophyletic group of genes comprise some of the TFs included in Table 1, e.g. HvMYBS3 (barley), LeGAMYBL1 (tomato), different groups of OsGAMYB, TaGAMYB (wheat), and ScGAMYB (rye).

The rest of MYBs mentioned in this work are placed in the subfamily VIII, the most functionally and structurally diversified among land plants. More than half of the R2R3-MYB TFs within land plant species belonged to this subfamily VIII-E. This subfamily underwent a process of duplication and subsequent expansion that reflected the subdivision into multiple subclades. Among the most relevant are MYB14, MYB30 and MYB96 orthologs. This sublineage, highly diversified in monocots and eudicots, are included within the subclade VIII-D, and seed development- related are distributed into two sublineages, β (MYB30 and MYB96) and γ (MYB14), named as subgroup 1 and 2 by Dubos classification. Finally, the remaining group of R2R3-MYB seed genes reviewed here fall into two distinct subgroups within the most diversified subclade VIII-E. Among these, MYB62 and MYB116 cluster within the δ sublineage together with TT2 TF genes. TT2, which is associated with seed coat development, dormancy, and germination, has a well-characterized regulatory mechanism, as described in the previous section.

In sum, phylogenetic mapping highlights a clear division of labor within the R2R3-MYB family. Ancient GA-responsive clades drive the execution of germination, whereas younger, ABA-associated clades modulate dormancy depth and environmental response. This evolutionary layering provides a mechanistic framework for understanding how conserved developmental programs are flexibly tuned to diverse ecological niches.

## Future perspectives and challenges for R2R3-MYB TFs in seed biology

Progress in understanding seed dormancy and germination has not advanced in parallel with the substantial efforts made by numerous highly competent research groups. This slow pace is attributable to the considerable variability among species, the historical lack of advanced molecular tools applied to plants, the high degree of compartmentalization characteristic of plant cells and of seeds themselves, the multifactorial and context-dependent nature of hormonal and environmental regulation, the inherent biological complexity of both processes—encompassing intricate hormonal networks, transcriptional regulators, and environmental cues that are challenging to integrate—and the methodological difficulties associated with studying highly dynamic, time-dependent developmental stages.

This review provides an extensive and detailed compilation of data on R2R3-MYBs, one of the largest TF families in plants. However, their seed-specific roles remain only partially understood. MYB factors represent highly promising tools for deepening our understanding of SLC, as they play central roles in regulating key processes, such as dormancy, germination, and early seedling growth. To fully leverage the insights provided by MYB proteins, it is essential to combine them with complementary approaches to validate findings and build a more integrated understanding of the complex regulatory networks controlling seed development. Integrative omics approaches, including transcriptomics, proteomics, and epigenomics, coupled with advanced imaging and single-cell analyses, may provide unprecedented resolution of MYB functions in specific seed tissues and developmental stages. In this context, the future incorporation of spatial multi-omics and high-throughput phenotyping platforms may further refine our capacity to map MYB activities in vivo with both temporal and spatial precision. Below, a series of approaches are outlined that should be implemented—while also taking advantage of the wide range of methodologies currently applied in animal systems—to help elucidate why plant cells harbor such a remarkably large repertoire of R2R3-MYB proteins.

Using existing knowledge from several model plant species on individual R2R3-MYB members, including genome-wide identification as well as evolutionary and expression analyses, a key priority is to determine which of these factors have demonstrable roles in shaping the seed life cycle. These processes include zygotic embryogenesis, seed dormancy, and germination.

In other words, several effects of R2R3-MYB transcription factors have been demonstrated in different tissues and organs within a given species; however, their involvement in the seed life cycle has not yet been established. For example, although the role of MYB96 in cuticular wax physiology has been documented in multiple plant tissues, its function in specific seed compartments remains unknown. This represents an important knowledge gap that should be addressed in future studies. What functions might the uncharacterized R2R3-MYB TFs have, not only in model plants, but also in other plant species? This requires clarifying the downstream genes they regulate, the specific seed compartments in which they act, and the temporal dynamics of their activity. In other words, it is crucial to undertake comprehensive functional studies of those R2R3-MYB genes whose involvement in the SLC has already been fully or partially suggested. Likewise, epigenetic variation—particularly DNA methylation and histone modifications—has a profound influence on the expression and function of MYB genes. For this reason, this largely unexplored area should be regarded as a research topic of high interest. Particular emphasis should be placed on determining how epigenetic states are reset or maintained across seed generations, and on whether MYB-dependent regulatory modules contribute to transgenerational plasticity in seed traits. Functional studies should also consider potential redundancy and compensatory mechanisms among MYB family members, which may mask phenotypic effects in single-gene perturbations.

The impact of functional manipulations on the networks directly involved in the final steps of zygotic embryogenesis is decisive. This should include not only the generation and characterization of loss and gain-of-function mutants, but also the identification of physical interactions with components of the signaling pathways that orchestrate seed development, dormancy, and germination. Equally important is the detailed examination of the phenotypic consequences arising from such perturbations, which will provide essential clues for disentangling the highly complex regulatory framework that underpins the seed life cycle. Here, integrating computational modeling and network inference approaches may help predict emergent behaviors of MYB-centered regulatory circuits, providing testable hypotheses that can guide future functional experimentation.

Finally, translating these insights into crop improvement requires careful evaluation of potential pleiotropic effects to ensure agronomic viability and environmental safety. In parallel, an important future challenge will be to harness this knowledge for its safe and efficient incorporation into genetic improvement and biotechnological initiatives. Developing genome-editing strategies that enable precise, tissue-specific, or inducible modulation of MYB activity will be essential, as will the establishment of standardized phenotyping pipelines to assess the impact of MYB manipulation on seed performance under realistic field conditions. Ultimately, bridging fundamental MYB biology with translational applications will determine the practical relevance of these TFs for enhancing seed quality, resilience, and yield.

## Data Availability

Not applicable. No new data were generated or analyzed in this review article.

## Abbreviations

ABA: Abscisic acid
ACN: Anthocyanin
BR: Brassinosteroid
DOG1: Delay of germination
FLAVs: Flavonoids
GA: Gibberellin
ISR: Induced systemic resistance
Jas: Jasmonates
PHS: Pre-harvest sprouting
ROS: Reactive oxygen species
SAR: Systemic acquired resistance
SLC: Seed life cycle
TF: Transcription factor
TT1 and TT2: Transparent testa 1 and 2
Y2H: Yeast two hybrid
